# Invasive Congeners Differ in Successional Impacts across Space and Time

**DOI:** 10.1371/journal.pone.0117283

**Published:** 2015-02-06

**Authors:** Aaron S. David, Phoebe L. Zarnetske, Sally D. Hacker, Peter Ruggiero, Reuben G. Biel, Eric W. Seabloom

**Affiliations:** 1 Department of Ecology, Evolution, and Behavior, University of Minnesota, Saint Paul, Minnesota, United States of America; 2 Department of Integrative Biology, Oregon State University, Corvallis, Oregon, United States of America; 3 Department of Forestry, Michigan State University, East Lansing, Michigan, United States of America; 4 College of Earth, Ocean, and Atmospheric Sciences, Oregon State University, Corvallis, Oregon, United States of America; Trier University, GERMANY

## Abstract

Invasive species can alter the succession of ecological communities because they are often adapted to the disturbed conditions that initiate succession. The extent to which this occurs may depend on how widely they are distributed across environmental gradients and how long they persist over the course of succession. We focus on plant communities of the USA Pacific Northwest coastal dunes, where disturbance is characterized by changes in sediment supply, and the plant community is dominated by two introduced grasses – the long-established *Ammophila arenaria* and the currently invading *A. breviligulata*. Previous studies showed that *A. breviligulata* has replaced *A. arenaria* and reduced community diversity. We hypothesize that this is largely due to *A. breviligulata* occupying a wider distribution across spatial environmental gradients and persisting in later-successional habitat than *A. arenaria*. We used multi-decadal chronosequences and a resurvey study spanning 2 decades to characterize distributions of both species across space and time, and investigated how these distributions were associated with changes in the plant community. The invading *A. breviligulata* persisted longer and occupied a wider spatial distribution across the dune, and this corresponded with a reduction in plant species richness and native cover. Furthermore, backdunes previously dominated by *A. arenaria* switched to being dominated by *A. breviligulata*, forest, or developed land over a 23-yr period. *Ammophila breviligulata* likely invades by displacing *A. arenaria*, and reduces plant diversity by maintaining its dominance into later successional backdunes. Our results suggest distinct roles in succession, with *A. arenaria* playing a more classically facilitative role and *A. breviligulata* a more inhibitory role. Differential abilities of closely-related invasive species to persist through time and occupy heterogeneous environments allows for distinct impacts on communities during succession.

## Introduction

Ecologists have sought to understand the processes and mechanisms of succession for well over a century (e.g. [1–6]) with a focus on the way in which individual species can shape community composition through time. Early-colonizing species can have disproportionate influence on subsequent community succession due to founder effects (e.g. [7]), their ability to capitalize on available resources [8], or their positive responses to disturbance [9]. Invasive plant species in particular are often uniquely positioned to influence succession because many are well-adapted to the disturbed conditions which initiate succession [10–13]. In some cases, their high abundance or life-history strategies can also make them ecosystem engineers, capable of transforming habitats and having long lasting effects after they are gone [14], especially if they are capable of changing the disturbance regime themselves [10,13].

The extent to which early-colonizing invasive species alter succession may depend on how long they can persist in the community. Early-colonizing species tend to capitalize on low competition and high light availability during primary succession, and modify their environment to facilitate later-colonizing species before extirpation [3,4,8]. Species that are able to persist longer may inhibit establishment of later species [4]. For instance, invasive species could alter the dispersal processes of resident species [15] or directly compete with them for resources (e.g., [8]). Alternatively, invasive species may give way to later successional species or persist in the community but cease to limit establishment of colonizing species (see facilitation and tolerance models in [4,16]). Different invasive species that play inhibitory or facilitative roles will have distinct effects on the ecological succession of the community.

Invasive species could persist longer in communities if they are able to tolerate a wide range of environmental conditions experienced during succession (e.g. [17,18]). Species with broader environmental tolerances likely have wider distributions across spatial and successional gradients [19–22]. Wider environmental distributions could lead to greater impacts on resident species because the invasive species is able to invade more environments and persist within them for longer periods (e.g. [23]).

**Fig 1:**
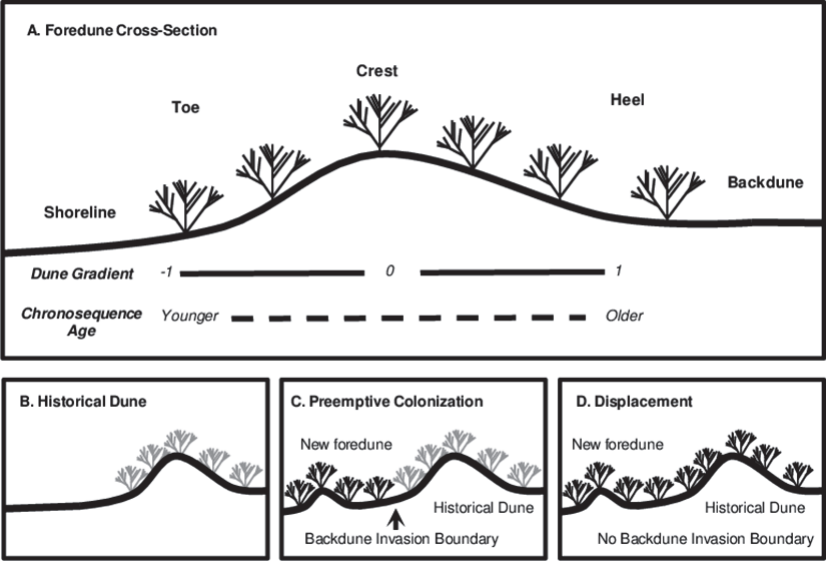
Foredune cross-section schematics. (A) Foredunes contain both spatial and temporal gradients along their cross-sections. Foredune consists of toe, crest, and heel moving from left to right. For the spatial dune gradient, quadrats were assigned values between-1 and 1 for their placement along the dune cross-section. Chronosequence age increased from the dune toe to heel (see main text for details). Shoreline and backdune are shown for orientation. (B) Historical dune consisting of only *Ammophila arenaria* (gray). (C) Invasion of historical dune by *A*. *breviligulata* (black) through preemptive colonization of a new dune. *Ammophila arenaria* retains its original population, and a backdune invasion boundary delineates the two species’ distributions along the dune. (D) Invasion of historical dune by *A*. *breviligulata* through displacement of *A*. *arenaria*. Note that there is no longer a backdune invasion boundary because the *A*. *arenaria* has been locally extirpated.

To better understand how invasive species influence resident communities during succession, we compared the distributions of two congeneric, invasive grasses across space and time in coastal dunes along the USA Pacific Northwest Coast. Coastal foredunes in this region, defined as linear ridges parallel and adjacent to the shoreline [24], contain rapidly advancing shorelines in many locations creating replicated chronosequences of herbaceous plant communities of up to 75 years (Fig. 1A). Furthermore, shoreline change rates vary widely across the region, making it is possible to separate the temporal effects of dune age from the purely spatial gradient created by proximity to the beach. Foredunes are invaded by two early-colonizing, introduced species of *Ammophila* beach grass—the longer established *A*. *arenaria* which is rapidly disappearing from northern foredunes, and the currently invading *A*. *breviligulata* which has potentially displaced it [25,26]. Pairs of invasive, congeneric species such as these can provide insights into the mechanisms and long-term consequences of invasion because they allow for comparisons between species occupying similar but not identical distributions (e.g., [26,27]. Whereas both grasses are important foredune stabilizers that can occur in nearly monodominant stands, *A*. *breviligulata* dunes are associated with lower plant diversity compared to *A*. *arenaria* dunes [26] and create foredunes of lower height [28], increasing the risk of coastal flooding [29]. Using chronosequence data we document shifts in dominance from *A*. *arenaria* to *A*. *breviligulata* as well as their associated herbaceous communities through successional time. *Ammophila breviligulata* may outcompete *A*. *arenaria* on foredunes [30], but it is not yet clear whether the former invades by preemptively colonizing new foredunes created by sand deposition or whether it can displace established *A*. *arenaria* in foredunes and historical dunes further inland (Fig. 1B-D). Understanding the distributions of the two species may provide insight into how *A*. *breviligulata* invades. Here we investigated the distributions of two high impact invasive species over two decades to ask the following questions: First, we asked how distributions of these two invaders differed across space and time. We hypothesized that the more recent invader *A*. *breviligulata* occupied a wider spatial distribution across the dune and persists in later-successional habitats, which allowed it to replace *A*. *arenaria* on foredunes [26]. Second, we asked how these distributional changes corresponded to impacts on resident communities. We hypothesized that the more broadly distributed species would also be associated with lower total species richness and native species abundance across space and through time. To test these hypotheses, we first used a chronosequence study to determine the distribution of both *Ammophila* species along successional and spatial environmental gradients. We then asked whether the *Ammophila* species differed in their associations with native plant cover, total species richness, and soil properties along spatial and successional environmental gradients. Additionally, we resampled transects after 21 years to determine whether *A*. *breviligulata* invasion had led to a predictably wider spatial distribution in *Ammophila* across dune cross-sections and whether *A*. *breviligulata* had displaced *A*. *arenaria* in backdunes.

## Materials And Methods

### Study System

Coastal dune systems are ideal for understanding how invasive species may affect succession of plant communities because they contain strong spatial gradients in community composition and physical characteristics [1]. We define the ‘dune gradient’ as the spatial gradient beginning at the start of the vegetation (occurring at or slightly seaward of the dune toe) and extending to the heel [25] (Fig. 1A). From toe to heel, there are often increases in nutrient availability and moisture, and decreases in wind, sand scour, and salt spray [11,31]. Plant communities along dune cross-sections typically begin as a few early-colonizing plants (e.g., *Ammophila*, *Cakile*) that begin stabilizing the sand, and gradually accumulate other grass, forb, and occasionally shrub species towards the dune heels [32]. In the backdune, the grass and forb dominated communities give way to shrubs and forest [32]. For the chronosequence and decadal studies described below, we focus on the relatively-early succession of the herbaceous community in the foredune, and do not address the long-term successional processes associated with forest development in the backdune. Succession of herbaceous foredune communities may influence the populations of several plant species endemic to dunes, and as well as animals such as the endangered Western Snowy Plover [26,33]. Because new dunes are continuously being formed via sand deposition, herbaceous foredune plant communities persist through time, even if individual dunes eventually become forested.

In some of the USA Pacific Northwest beaches in Washington and Oregon, the shoreline is expanding seaward with the deposition of wave and wind delivered sand [34]. This phenomenon creates a chronosequence along dune cross-sections in which inland areas are older than more seaward and recently-formed areas [11] (Fig. 1). Variation in sand supply across the region [26,34] may lead to differences in foredune shape and age. From central Oregon north, high sand supply leads to wider, shorter, and younger foredunes created by the recently deposited sand and are typically dominated by *A*. *breviligulata* [26,28,35]. In contrast, from central Oregon south, sites are typically dominated by *A*. *arenaria* and experience relatively low sand supply which leads to narrower, taller, and older foredunes, the result of small amounts of sand deposition in one location over many years [26,28,35].

In response to sand deposition, both *Ammophila* species advance into newly created bare sand habitat and build foredunes through sand capture [26,28]. The European beach grass, *Ammophila arenaria*, was introduced for dune stabilization in the early 1900’s to the US Pacific Northwest Coast, and the American beach grass, *A*. *breviligulata*, was later introduced to northern Oregon in 1935 from the Eastern USA [36]. *Ammophila breviligulata* has since spread along the coast throughout the region, sharply reducing *A*. *arenaria* in Washington and northern Oregon dunes while not yet reaching southern Oregon [25,26,28]. Both beach grass invaders are superficially similar in morphology and growth form, and are the primary species involved with sand capture and creation of foredunes due to their high tiller density [25,26,28] However, their distinct growth forms create different shaped dunes as a result [25,26,28].

### Chronosequence Study

We investigated the distributions of *Ammophila* species abundance, native species abundance, and total plant species richness along dune cross-sections from 52 unique transects surveyed in 2006 and 2009 (32 transects surveyed in both years, 2 surveyed only in 2006, 18 surveyed only in 2009; S1 Appendix). Analysis of total richness allows for an understanding of the accumulation of species through space and time, while that of native plant abundance addresses how *Ammophila* specifically impacts the native vegetation. Plant community composition and dune morphology data were collected along transects running perpendicular to the shoreline, from the foredune toe to its heel in the backdune (see [25,26] for transect methodology) (S1 Appendix). Every 5m along each transect, we visually estimated percent cover of every plant species present within a 50x20cm quadrat, and transects ranged from 40m-325m in length. Permission to access field sites was granted by United States Forest Service, United States Fish and Wildlife, Willapa Bay Wildlife Refuge, Washington State Parks and Recreation Commission, Oregon Parks and Recreation Department, Scott McKenzie Ranch, and Lane County Park.

To determine underlying soil properties of foredunes, we collected soil samples from *A*. *arenaria* and *A*. *breviligulata* dominated dunes at three locations along foredunes—the toe, crest, and heel (Fig. 1A). Soil cores (20.5 long x 2.5 cm diameter) were collected from 21 sites varying in dominant *Ammophila* species, shoreline change rate, and latitude in 2012 (S1 Appendix). For each location within a site, three replicate samples were taken. Soils were stored at-20°C following collection, air-dried at 60°C, homogenized within locations at a site, and sieved through a 500μm mesh to remove debris before analysis. We obtained the following soil properties: percent C, percent N, concentrations of P, K and Na, organic matter, pH, cation exchange capacity.

For each transect surveyed, we also estimated shoreline change rate, a measure of how much habitat has been formed from sand deposition (positive) or lost due to erosion (negative). Shoreline change rate was calculated from two endpoints: 1) proxy-based shorelines derived from 1950–60s era aerial photographs; and 2) datum-based lidar-derived shoreline positions from 2002 [34].

For vegetation transects with a net positive shoreline change rate, we used the estimated shoreline change rate to interpolate the approximate ages of the locations of each quadrat after adjusting for the average distance between the shoreline and the start of the vegetation. This adjustment was made to correct for age differences between the shoreline location where the shoreline change rate was measured and the start of the vegetation where transects began. We used a natural log transformation of chronosequence age in all analyses to avoid placing too much weight on older quadrats whose age estimates could be more prone to error. In our dataset, quadrats in *A*. *breviligulata* dominated dunes (range: 4.00–75.00 years; mean: 20.22) were younger in chronosequence age than those in *A*. *arenaria* dominate dunes (range: 5.00–55.00 years; mean: 23.47) (t-test d.f. = 133, t = 3.23, p <0.001). We defined the dune gradient for each quadrat as the standardized location of each quadrat on the dune ranging from-1 to 1, where-1, 0, and 1 refer to the toe, crest, and heel, respectively (Fig. 1A). While chronosequence age and dune gradient were positively correlated (Pearson’s r = 0.337), most of the variance in age was uncorrelated with distance allowing for independent test of chronosequence age and dune gradient. We used the proportional cover of each *Ammophila* species per transect to categorize transects as either dominated by *A*. *arenaria* or *A*. *breviligulata* (proportion of dominant species cover > 0.5). For each quadrat, we calculated *Ammophila* cover as the sum of the raw percent cover of both *Ammophila* grasses, native cover as the sum of all native species cover, and species richness as the number of species present (excluding *Ammophila*). Total cover per quadrat could exceed 1due to overlapping plant canopies.

To analyze the effects of the dune gradient and chronosequence age on *Ammophila* cover, native cover, and richness, we used hierarchical Bayesian models to determine the significant factors associated with our response variables. We modeled the logit-transformed *Ammophila* cover per quadrat as a function of the shoreline change rate, dominant *Ammophila* species, chronosequence age, dune gradient, and their interactions and quadratic relationships, along with the random effects of transect and year (S2 Appendix). Similarly, we modeled logit-transformed native cover and untransformed species richness (Poisson distribution) per quadrat as a function of the same predictor variables as well as *Ammophila* cover (S2 Appendix). A strong effect of dominant *Ammophila* species × chronosequence age was taken as evidence that the two *Ammophila* species differed in their effects through time on the response variable. Likewise, a strong effect of dominant *Ammophila* species × dune gradient was taken as evidence that the two *Ammophila* species affected response variables differently along the dune gradient. For all models, continuous predictor variables were normalized to allow for comparison of estimates. We used a normal prior for all fixed parameters [37]. For the random effects of transect and year, we estimated random intercepts with hyperparameters for the mean and standard deviation using normal and uniform priors, respectively [37]. Initial parameter values were drawn from the normal distribution, and the initial variance was drawn from the lognormal distribution [37]. We ran 50,000 iterations with 500 iterations of burn-in and a thinning rate of 2. We assessed model convergence using 10 independent chains and the Gelman-Rubin diagnostic statistic. Analyses were conducted using the *R2jags* package [38] in R Version 3.0.2 [39]. To visualize results, we created contour plots using the predicted values of generalized linear models with the form *response variable* ∼ *chronosequence* + *dune gradient*.

For soils, we used analysis of variance to analyze the effect of the dominant *Ammophila* species and the effect of location within the dunes on each soil property.

### Decadal study

Using plant community transect surveys from southwestern Washington, USA, we investigated how *Ammophila* cover and plant species richness changed over a 21-year period (1988–2009), during which time *A*. *breviligulata* became the dominant species at all transects (S1 Appendix). Specifically, we compared how transects that have switched in dominance from *A*. *arenaria* in 1988 to *A*. *breviligulata* by 2009 differed from those that were already *A*. *breviligulata*-dominant in 1988 and remained so in 2009. We also considered how such switches in *Ammophila* dominance varied in their effects across the dune gradient.

The decadal dataset consisted of 34 transects along the Washington coast that all had some degree of *A*. *breviligulata* invasion (S1 Appendix). Because *A*. *breviligulata* had become the dominant species in all transects by 2009, we classified transects as either ‘switched’ from *A*. *arenaria* to *A*. *breviligulata* dominated or ‘established’ by *A*. *breviligulata* in 1988 and remaining so in 2009. We calculated the proportions of *A*. *arenaria* and *A*. *breviligulata* as the raw cover divided by the total *Ammophila* cover for each transect. We classified 7 transects as ‘switched’ in which there was substantial *A*. *arenaria* cover (proportions of *A*. *arenaria* ranging from 0.32–1.0 in 1988) and 27 transects as ‘established’ that were dominated by *A*. *breviligulata* (proportions of *A*. *breviligulata* ranging from 0.77–1.0 in 1988). To compare across transects, we standardized each quadrat’s location along the dune cross-section using the dune gradient index previously described.

We analyzed the relative *Ammophila* cover and plant species richness using mixed-effects models with linear contrasts. We assigned four groupings based on the factorial combination of *A*. *breviligulata* invasion (switched vs. established) and year (1988 vs. 2009). Our linear contrasts compared (1) overall effect of year, and the differences within (2) 1988 and (3) 2009 between switched and established transects. Response variables were modeled as response ∼ dune gradient + grouping + dune gradient × grouping and we used transect as a random intercept. To reduce observer sampling bias between years, we used the relative cover as the raw cover of *Ammophila* divided by the total plant cover for each quadrat. We used the logit-transformation of these relative cover values to meet the model assumptions. To analyze richness per quadrat, we fit a model of the number of species per quadrat with a Poisson error distribution. We calculated 95% confidence intervals for parameters using the Wald method [40].

### Backdune Invasion Boundary Study

In their 1988 surveys of the rapidly accreting Long Beach Peninusla, WA, USA, shoreline, Seabloom and Weidemann [25] found that while *A*. *breviligulata* dominated many of the foredunes, backdune areas were dominated by *A*. *arenaria*. They surveyed the location of this change in dominance, which we refer to as the backdune invasion boundary between *A*. *breviligulata* and *A*. *arenaria*. Though *A*. *breviligulata* has steadily increased its range latitudinally along the coast [26], this invasion has been thought to be restricted to foredunes where it preemptively colonizes newly formed, early successional dunes, whereas remnant *A*. *arenaria* stands in the later succesional backdunes are presumed to persist [25] (Fig. 1B-C). However, if *A*. *breviligulata* is capable of displacing *A*. *arenaria*, the latter should be reduced in older backdunes and an invasion boundary should no longer exist between *A*. *breviligulata* and *A*. *arenaria* dominated areas (Fig. 1D). We resurveyed the location of this boundary to determine whether *A*. *breviligulata* could invade and displace *A*. *arenaria* in stable backdune areas over the 23 year period between 1988 and 2011.

We resurveyed 16 of the 1988 transects and compared the backdune invasion boundaries to those previously observed by Seabloom and Wiedemann [25]. In 1988, Seabloom and Wiedemann [25] measured the distance from the toe of the dune to the point where the species composition changed from *A*. *breviligulata* to *A*. *arenaria* on Long Beach Peninsula, WA. Using aerial photos, we determined the GPS coordinates of these transects and the 1988 location of the compositional changes in herbaceous backdunes. In 2011, we visually determined whether such a compositional change had occurred by resurveying each transect extended landward to forest or human development.

## Results

### Chronosequence Study

**Fig 2:**
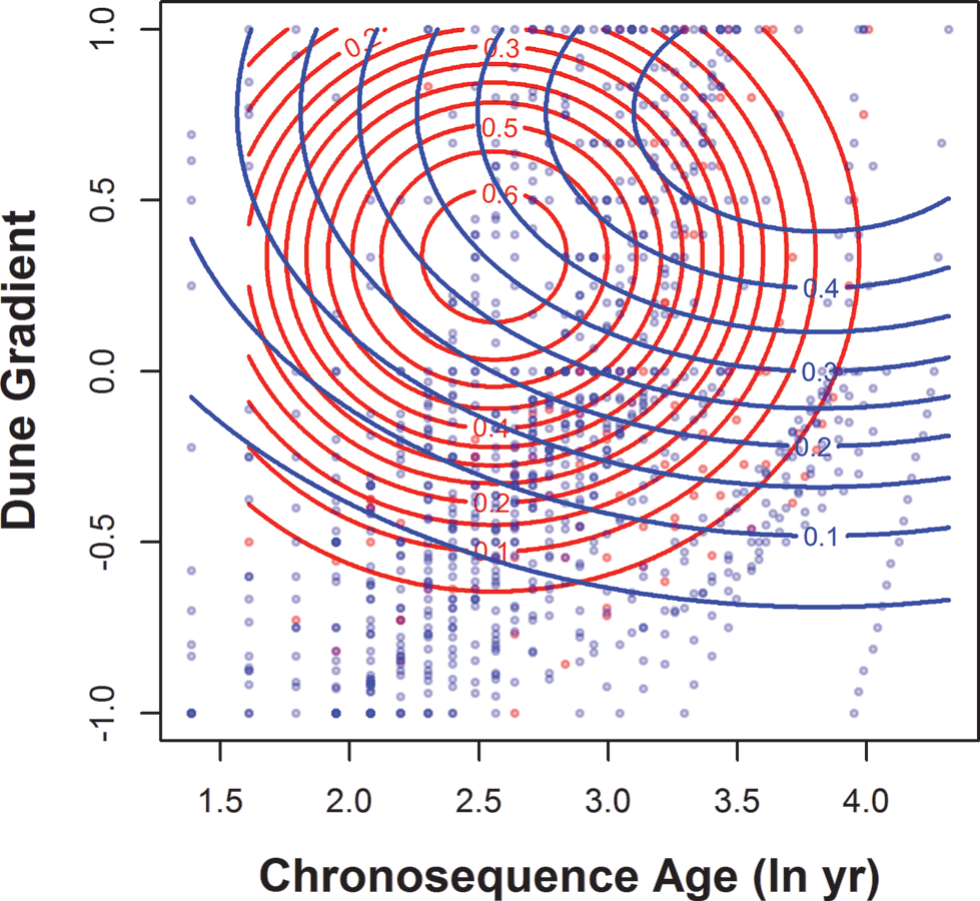
Contour plot of *Ammophila* cover shows distinct species distributions across chronosequence ages and dune gradient. Contours show areas of increasing cover across both gradients. Contours were created using predicted values of generalized linear models (see main text for details). Chronosequence ages were calculated for each quadrat using aerial photos and shoreline change rates (see main text for details). Dune gradient was calculated for each quadrat based on its standardized location along dune cross sections with the toe, crest, and heel at-1, 0, and 1, respectively. Each point on figure refers to the chronosequence age and dune gradient for an individual quadrat. Sites dominated by *A*. *arenaria* are shown in red, and those from *A*. *breviligulata* sites shown in blue.

*Ammophila breviligulata* occupied a wider span of chronosequence age and dune gradient compared to *A*. *arenaria* (Fig. 2; Bayesian model Gelman-Rubin diagnostic statistic < 1.01 for all parameters). *Ammophila* cover towards the heel of the dune was higher in *A*. *breviligulata* dunes than in *A*. *arenaria* dunes as evidenced by the significant quadratic effect of dominant *Ammophila* species × dune gradient (S3 Appendix). *A*. *breviligulata* also had higher cover in quadrats of older chronosequence age (S3 Appendix) as evidenced by the significant effect of dominant *Ammophila* species × chronosequence age^2^. *Ammophila* abundance was generally lower with higher shoreline change rate (S3 Appendix).

**Fig 3:**
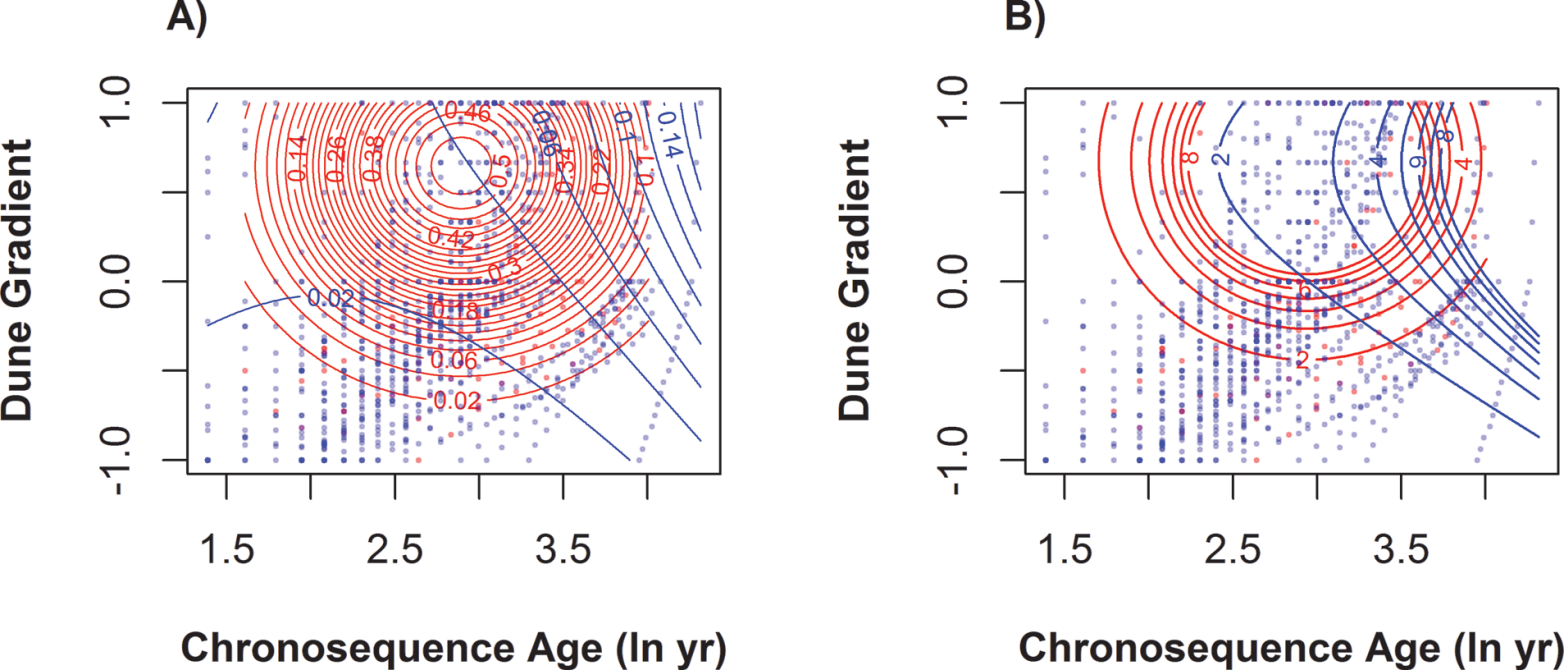
Contour plot of (A) native cover and (B) species richness shows differences in distribution across across chronosequence ages and dune gradient in dunes of different *Ammophila* dominance. Contours show areas of increasing cover or richness across both gradients. Quadrats from sites dominated by *A*. *arenaria* are shown in red, those from *A*. *breviligulata* sites shown in blue. See main text and Fig. 2 caption for additional details.

The distribution of native cover differed between dunes dominated by *A*. *breviligulata* versus *A*. *arenaria* along both the dune gradient and chronosequence (Fig. 3A). We found a significant 3-way interaction between the dominant *Ammophila* species, chronosequence age, and the dune gradient, indicating that all three factors contribute to native cover. Higher total *Ammophila* cover and the presence of *A*. *breviligulata* were both associated with less native cover. Native cover was best explained by the dominant *Ammophila* species × dune gradient interaction; *A*. *arenaria* dunes had higher native cover closer to the shore (i.e., lower dune gradient indices) relative to *A*. *breviligulata* dunes (S4 Appendix). Similarly, *A*. *arenaria* dunes had higher native cover in younger communities (lower chronosequence ages) compared to *A*. *breviligulata* dunes.

The distribution of total species richness also differed between *A*. *arenaria* and *A*. *breviligulata* dunes (Fig. 3B). There was no significant main effect of the dominant *Ammophila* species on richness (S4 Appendix), although richness increased with chronosequence age and along the dune gradient. *A*. *arenaria* dunes tended to have higher richness at lower chronosequence ages than *A*. *breviligulata* dunes. In contrast, *A*. *breviligulata* dunes had higher richness at higher chronosequence age. Richness was not associated with *Ammophila* cover.

Soil properties differed according to the dominant *Ammophila* specie*s* and the location (toe, crest, heel) along the dune gradient (S5 Appendix; S6 Appendix). Percentage of soil C, N and organic matter generally increased moving inland from the beach, and were higher in dune heels of *A*. *breviligulata* dunes than in those of *A*. *arenaria* dunes. Concentrations of P, K, and Na and cation exchange capacity generally decreased moving inland from the beach and varied between the dunes with different *Ammophila* species. Soil pH decreased from toe to heel more in *A*. *breviligulata* than in *A*. *arenaria* dunes.

### Decadal Study

**Fig 4:**
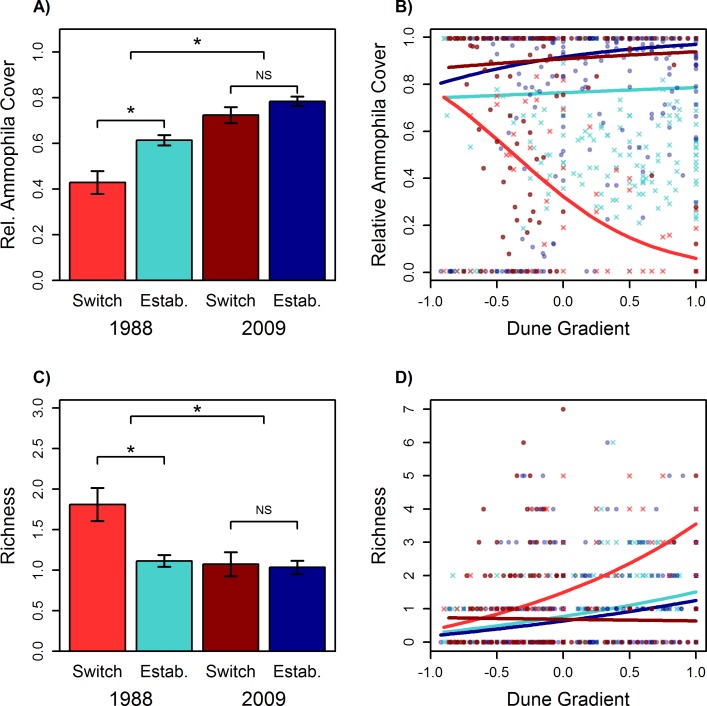
Changes in *Ammophila* cover and plant richness over a 21-year period from 1988 to 2009. ‘Switch’ refers to transects that had substantial *A*. *arenaria* in 1988 and were dominated by *A*. *breviligulata* in 2009. “Estab.’ refers to transects that were dominated by *A*. *breviligulata* in both 1988 and 2009. (A) Mean relative *Ammophila* cover ± S.E.; (B) Relative *Ammophila* cover across the dune gradient; (C) Mean plant richness ± S.E.; (D) Plant richness across the dune gradient. Light red denotes switched transects in 1988; light blue denotes established transects in 1988; dark red denotes switched transects in 2009; dark blue denotes established transects in 2009. Three separate linear contrasts shown at different heights in (A) and (C), stars signify significance (p < 0.05), NS signifies not significant. For (B), there was a significant difference in *Ammophila* cover between switched and established transects in 1988 but not in 2009. For (D), there were no significant differences in richness along the dune gradient within either 1988 or 2009. See Table 1 for full models.

Total *Ammophila* cover increased significantly from 1988 to 2009, indicating that it had increased its relative dominance in the plant community (Table 1; Fig. 4A). Transects that switched from *A*. *arenaria* to *A*. *breviligulata* had significantly less total *Ammophila* cover than established transects in 1988, but there was no difference in 2009 when all transects were dominated by *A*. *breviligulata*. This result can be attributed to lower *Ammophila* cover in the backdune of switched transects compared to the established transects in 1988, but again there was no difference in 2009 (Table 1; Fig. 4B).

**Table 1:** Results from the decadal study using linear mixed models with independent linear contrasts.

Source	*Ammophila* Cover		Species Richness	
	Estimate	Wald 95% CI	Estimate	Wald 95% CI
(Intercept)	1.26	(0.85, 1.67)	0.00	(-0.22, 0.21)
Dune gradient	-0.24	(-0.89, 0.41)	0.79	(0.62, 0.96)
2009 vs. 1988	1.04	(0.72, 1.37)	-0.10	(-0.19, -0.01)
Switched sites vs. established sites (within 1988)	-0.97	(-1.53, -0.41)	0.26	(0.03, 0.5)
Switched sites vs. established sites (within 2009)	-0.08	(-0.56, 0.39)	0.12	(-0.12, 0.35)
dune gradient (2009) vs. dune gradient (1988)	0.79	(0.14, 1.45)	0.01	(-0.17, 0.18)
dune gradient (switched sites) vs. dune gradient (established sites) (within 1988)	-1.15	(-2.17, -0.13)	0.04	(-0.21, 0.29)
dune gradient (switched sites) vs. dune gradient (established sites) (within 2009)	-0.51	(-1.32, 0.31)	-0.07	(-0.31, 0.17)

Sites were categorized as ‘switched’ (*A*. *arenaria* dominated in 1988 and *A*. *breviligulata* dominated in 2009) or ‘established’ (*A*. *breviligulata* dominated in both 1988 and 2009). Relative *Ammophila* cover was logit-transformed and analyzed with Gaussian error distribution, and species richness was analyzed using a Poisson error distribution. Estimates for individual contrasts and Wald 95% confidence intervals are shown. Independent contrasts tested (1) the effect between 1988 and 2009, (2) the effect between switched and established sites in 1988 when sites were either *A*. *arenaria* or *A*. *breviligulata* dominated, and (3) the effect between switched and established sites in 2009 when all sites were *A*. *breviligulata* dominated. Terms are bolded as significant when the CI did not contain 0.

Plant species richness significantly decreased from 1988 to 2009 (Table 1; Fig. 4C). Switched transects had significantly higher richness than the established transects in 1988, but there were no differences between switched and established transects in 2009. We found no significant differences along the dune gradient for any of the linear contrasts (Table 1, Fig. 4D).

### Backdune Invasion Boundary Study

We found no evidence of a boundary between *A*. *breviligulata* and *A*. *arenaria* dominated communities in backdunes sampled in 2011. Of the 16 resurvey transects, 10 of the 1988 backdune invasion boundaries were now located beyond the foredune and were forested (n = 6) or developed (n = 4). All of the remaining transects (n = 6) were dominated by *A*. *breviligulata*. We found isolated patches of *A*. *arenaria* in only 4 transects.

Datasets and metadata for all studies as well as R script for the Bayesian analyses may be found in S7 Appendix.

## Discussion

The extent to which invasive species persist through time and tolerate varying spatial environments may determine their impact on succession. We have shown that while closely related invasive species overlap in space and time, their resulting distributions are associated with differential native plant cover and richness. Specifically, we found that the newer invader, *A*. *breviligulata* persisted at higher abundance in the backdunes and at later chronosequence ages than the established invader, *A*. *arenaria*. These findings show that *A*. *breviligulata* occupies a wider distribution than *A*. *arenaria*, and has the potential to have broader impacts on plant species richness, native cover, and soil nutrients through time and space. Furthermore, over the past two decades *A*. *breviligulata* invasion into foredunes previously dominated by *A*. *arenaria* has led to a predictable increase in *Ammophila* cover in the backdune, though richness did not significantly differ along dune cross-sections in sites of different invasion history. Finally, we observed that *A*. *breviligulata* dominates in backdune areas where a boundary between the two species once existed, suggesting that *Ammophila breviligulata* has displaced *A*. *arenaria* and has the potential to limit native cover and richness beyond the foredune. Our results provide evidence that two closely related invasive species occupy similar yet distinct distributions, with implications for their roles in succession. *A*. *breviligulata* may play a more inhibitory role in foredune succession than *A*. *arenaria*, and the former’s invasion may ultimately slow herbaceous successional processes.

Invasive species that are widespread and can tolerate a range of conditions may have the greatest impact on succession. They may inhibit colonizing species for long periods of time [4], and thus slow the recovery of plant communities after disturbances [41]. They may also remove spatial refugia for resident species, potentially causing reductions in population growth rates [42,43]. Several mechanisms could explain why *A*. *breviligulata* has a wider distribution and potentially larger impacts on species richness than *A*. *arenaria*. *Ammophila breviligulata* may be a superior competitor for resources [30], or could also be a faster colonizer and preemptively colonize new habitat. Species-specific differences in morphology and sand capture ability may favor *A*. *breviligulata* in the dune heels and backdunes and allow it to displace *A*. *arenaria* [26,30]. *Ammophila arenaria*, which tends to grow vertically in response to sand deposition, depends much more on sand burial to achieve high growth than does *A*. *breviligulata* [28]. Therefore, *A*. *breviligulata* could have an advantage in backdunes with low sand burial. Finally, *A*. *breviligulata* could have more favorable interactions with symbiotic organisms such as arbuscular mycorrhizae [44] or fungal endophytes [45] found in dune systems or experience a greater benefit from natural enemy release from pathogens such as nematodes [46,47], particularly if these organisms vary in their host specificity or distribution along dunes.

*Ammophila breviligulata*’s strong impact on native cover and richness could be attributed to feedbacks between vegetation and biophysical processes that alter the physical features of a landscape through time [28,48]. Both *Ammophila* species build dunes by capturing sand and changing the topography of their habitat [28], therefore the two species may uniquely alter dune morphologies and the resulting local dune conditions. Spatial gradients may impose an environmental filter that influences where certain species are able to colonize during succession [49,50]. This potentially creates distinct local environmental conditions between dunes dominated by the two *Ammophila* species in terms of soil properties (S6 Appendix), as well as alter salt spray and wind exposure [31]. Interestingly, soil in dune heels dominated by *A*. *breviligulata* was more characteristic of late dune succession (e.g., higher C and N) than *A*. *arenaria* soils [11,51] despite having lower native cover. It is possible that plant-soil feedbacks differ between the *Ammophila* species, leading to higher nutrient content in *A*. *breviligulata* soils. Additionally, higher nutrient content simply could have a minimal effect on plant communities given that nutrient levels were generally low, and could suggest that traits such as response to disturbance are more important than competition for resources such as nitrogen (e.g. [52]). Positive responses to disturbed environments (e.g. higher wind, sand burial [53]) could be a mechanism by which the two species of *Ammophila* reduce establishment of colonizing species. For instance *A*. *arenaria* may rely on rapid sand burial to displace colonizing native species the foredune toe and crest (e.g. [54]), but low sand supply in dune heels would lessen its ability to do so. The horizontal growth pattern of *A*. *breviligulata* [26,28] could also contribute to lower richness in dune toes by allowing *A*. *breviligulata* to rapidly create new dunes devoid of species that have yet to colonize.

Furthermore, *A*. *breviligulata* may not necessarily differ from *A*. *arenaria* in its direct interactions with the herbaceous plant community, but rather its wider distribution may augment its ecological effects. For instance, both *Ammophila* species may inhibit the establishment of colonizing species by leaving fewer unconsumed resources [8,55], and high *Ammophila* cover and tiller densities [26,28] may also reduce light availability for colonizing species. In our study, we found that the two *Ammophila* species were associated with similar native cover and richness in younger dunes, and only found differences in older dunes where *A*. *arenaria* cover was reduced. *Ammophila* species’ interactions with herbaceous plant communities could be likely mediated via differences in biophysical dune feedbacks [28,30], and the *Ammophila* species’ respective abilities to persist in older, landward parts of the dune.

Following the distributions of invasive species over space and time may provide insights into the classical successional roles they play (*sensu* [4]). Specifically, our results suggest that the invasion of the more widely-distributed *A*. *breviligulata* has shifted the role of *Ammophila* on foredunes from that of facilitator to inhibitor. *Ammophila arenaria* may play a facilitative role as an early colonizer that gives way to later colonizing species through time (*sensu* [4]). In addition to occupying a narrower distribution, *A*. *arenaria* was excluded from herbaceous backdune communities, likely due to displacement from *A*. *breviligulata* on the shoreside and forest encroachment on the landward side. In contrast, *A*. *breviligulata* may play a more inhibitory role (*sensu* [4]), maintaining dominance over longer periods of successional time and reducing native cover and richness. The shift from a more facilitative to inhibitory role of the dominant species has important consequences for how subsequent species establish, interact, and ultimately form communities. Invasive species, particularly those present at the initiation of succession, that are able to persist longer and tolerate more spatially-heterogeneous environments may have the greatest impact on the succession of resident communities. We have shown that two seemingly similar invasive species can differ in these regards and play distinct roles in succession.

## Supporting Information

S1 Appendix. (DOCX)

S2 Appendix. (DOCX)

S3 Appendix. (DOCX)

S4 Appendix. (DOCX)

S5 Appendix. (DOCX)

S6 Appendix. (DOCX)

S7 Appendix. (ZIP)
