# Supplementary material for: Invasive Congeners Differ in Successional Impacts across Space and Time
Source: PLoS One. 2015 Feb 6;10(2):e0117283. doi: 10.1371/journal.pone.0117283 (PMC4319750; doi:10.1371/journal.pone.0117283)
Supplement: S1 Appendix. — (DOCX) [file pone.0117283.s001.docx]

**Supplementary Information 1** Transects used for chronosequence study, soil study, and decadal study. The proportion of *A. breviligulata* and the proportion of *A. arenaria* (not shown) sum to 1.

| **Transect** | **Littoral Cell** | **UTM Northing (Zone 10T)** | **Shoreline Change Rate (m/yr)** | **Prop. *A. breviligulata*** | | | | **Chronosequence Study**  **(2006 and 2009)** | **Soil study**  **(2012)** | **Decadal study**  **(1988-2009)** |
| --- | --- | --- | --- | --- | --- | --- | --- | --- | --- | --- |
|  |  |  |  | 1988 | 2006 | 2009 | 2012 |  |  |  |
| **GH82** | Columbia River | 5189593 | 1.7 | 0 | 0.68 | 0.56 |  | x |  | x |
| **GH18** | Columbia River | 5189352 | 1.5 | 0 | 0.55 | 0.8 |  | x |  | x |
| **GH17** | Columbia River | 5189059 | 1.6 | 0 | 0.79 | 0.98 |  | x |  | x |
| **GH09** | Columbia River | 5187765 | 0.6 | 1 | 0.97 | 1 |  | x |  | x |
| **GH06** | Columbia River | 5186818 | -0.1 | 0.89 | 0.83 | 1 | 1 |  | x | x |
| **GH05** | Columbia River | 5183438 | 1.1 | 0.93 | 0.85 | 1 | 1 | x | x | x |
| **GH13** | Columbia River | 5183283 | 1.2 | 0.86 | 1 | 0.86 |  | x |  | x |
| **GH01** | Columbia River | 5182372 | 2.2 | 0.94 | 1 | 1 |  | x |  | x |
| **GH12** | Columbia River | 5180716 | 6.1 | 0.91 | 1 | 1 |  | x |  | x |
| **GH07** | Columbia River | 5179641 | 11.5 | 1 | 1 | 1 |  | x |  | x |
| **GH03** | Columbia River | 5179506 | 11.1 | 0.95 | 1 | 1 |  | x |  | x |
| **GH11** | Columbia River | 5179023 | 9.7 | 0.77 | 1 | 1 | 0.79 | x | x | x |
| **GH04** | Columbia River | 5177364 | -2.1 | 0.83 | 1 | 1 |  |  |  | x |
| **GH20A** | Columbia River | 5177061 | -2.6 | 0.21 | 0.85 | 1 |  |  |  | x |
| **GH20** | Columbia River | 5176872 | -8.6 | 0.1 | 1 | 0.87 |  |  |  | x |
| **LBP06** | Columbia River | 5163832 | 4.6 |  |  | 0.99 |  | x |  |  |
| **LBP05** | Columbia River | 5163508 | 4.8 |  |  | 0.91 |  | x |  |  |
| **LB49** | Columbia River | 5163387 | 4.9 | 0.94 | 1 | 1 |  | x |  | x |
| **LBP07** | Columbia River | 5162745 | 4.3 |  |  | 1 |  | x |  |  |
| **LBP08** | Columbia River | 5162235 | 3.8 |  |  | 1 |  | x |  |  |
| **LB08** | Columbia River | 5161953 | 3.9 | 0.79 | 0.71 | 1 | 1 | x | x | x |
| **LB11** | Columbia River | 5161378 | 3.5 | 0.8 | 1 | 1 | 1 | x | x | x |
| **LB15** | Columbia River | 5160850 | 3.4 | 1 | 1 |  |  | x |  | x* |
| **LB1020** | Columbia River | 5156020 | 3.1 | 0.83 | 1 | 1 |  | x |  | x |
| **LB06** | Columbia River | 5154359 | 2.7 | 0.93 | 0.99 | 1 |  | x |  | x |
| **LB01** | Columbia River | 5154131 | 2.7 | 0.83 | 1 | 1 |  | x |  | x |
| **LB14** | Columbia River | 5151538 | 2.5 | 1 | 1 | 1 |  | x |  | x |
| **LB12** | Columbia River | 5151254 | 2.2 | 0.68 | 1 | 0.95 | 1 | x | x | x |
| **LB05A** | Columbia River | 5148967 | 3.8 |  |  | 1 |  | x |  |  |
| **LB05** | Columbia River | 5148482 | 3.8 | 1 | 1 | 1 | 0.58 | x | x | x |
| **LB02** | Columbia River | 5147332 | 4 | 1 | 1 | 1 | 0.72 | x | x | x |
| **LB03** | Columbia River | 5144911 | 5.1 | 1 | 1 | 1 |  | x |  | x |
| **LB09** | Columbia River | 5142778 | 5.3 | 0.64 | 1 | 1 |  | x |  | x |
| **LB07** | Columbia River | 5138252 | 5.9 | 0.84 | 1 | 1 |  | x |  | x |
| **LB35** | Columbia River | 5134045 | 4.8 | 1 | 1 | 1 |  | x |  | x |
| **LB36** | Columbia River | 5132039 | 3.8 | 1 | 1 |  |  | x |  | x* |
| **LB37** | Columbia River | 5130347 | 3.2 | 1 | 1 | 1 | 1 | x | x | x |
| **LB2842** | Columbia River | 5129357 | 1.6 | 1 | 1 | 1 |  |  |  | x |
| **LB32** | Columbia River | 5128977 | 1.5 | 1 | 1 | 1 |  | x |  | x |
| **EASTJETTY** | Columbia River | 5119128 | 0.8 |  |  | 0 | 0.71 |  | x |  |
| **FS02** | Columbia River | 5117392 | 0.2 |  | 0 | 0 |  | x |  |  |
| **IREDALE43** | Columbia River | 5114402 | 0.3 |  |  | 0 |  | x |  |  |
| **KIM44** | Columbia River | 5111068 | 0.6 |  |  | 1 | 1 | x | x |  |
| **RILEA45** | Columbia River | 5107099 | 3 |  |  | 1 |  | x |  |  |
| **FS03** | Columbia River | 5105783 | 2.5 |  | 0.59 | 1 | 1 | x | x |  |
| **FS01** | Columbia River | 5100274 | 1.8 |  | 0.92 | 1 |  | x |  |  |
| **DELRAY46** | Columbia River | 5099952 | 1.5 |  |  | 1 | 0.82 | x | x |  |
| **DELRAY46A** | Columbia River | 5099556 | 1.8 |  |  | 1 |  | x |  |  |
| **SEASIDE** | Columbia River | 5094724 | 0.8 |  |  | 0.91 |  | x |  |  |
| **NB04** | Rockaway | 5060094 | 1 |  |  |  | 0 |  | x |  |
| **NB03** | Rockaway | 5059198 | 0 |  |  |  | 0 |  | x |  |
| **PC01** | Neskowin | 5004502 | 0.3 |  | 0 | 0 |  | x |  |  |
| **SB03** | Newport | 4939934 | 1.9 |  | 0 | 0 |  | x |  |  |
| **SNJ03** | Coos Bay | 4874857 | 1.8 |  |  |  | 0 |  | x |  |
| **SSJ01** | Coos Bay | 4873812 | 2.5 |  |  |  | 0 |  | x |  |
| **SSJ03** | Coos Bay | 4872546 | 0.5 |  |  |  | 0 |  | x |  |
| **SILT03** | Coos Bay | 4857458 | 0.2 |  |  | 0 |  | x |  |  |
| **DONR06** | Coos Bay | 4855442 | 0.2 |  |  | 0 |  | x |  |  |
| **TKNR05** | Coos Bay | 4851487 | -0.4 |  |  | 0 | 0 |  | x |  |
| **TKNR04** | Coos Bay | 4851067 | 0.6 |  |  | 0 | 0 | x | x |  |
| **BANNR03** | Bandon | 4768995 | 0.7 |  |  | 0 |  | x |  |  |
| **BANNR06** | Bandon | 4768482 | 1.4 |  |  |  | 0 |  | x |  |
| **BANNR02** | Bandon | 4768307 | 1.6 |  |  | 0 |  | x |  |  |
| **BANNR01** | Bandon | 4766756 | 0.2 |  |  | 0 |  | x |  |  |
| **MCKNR02** | Port Orford | 4739800 | 0.5 |  |  | 0 |  | x |  |  |

*2006 data used in decadal study because 2009 data were unavailable
