# Supplementary material for: Invasive Congeners Differ in Successional Impacts across Space and Time
Source: PLoS One. 2015 Feb 6;10(2):e0117283. doi: 10.1371/journal.pone.0117283 (PMC4319750; doi:10.1371/journal.pone.0117283)
Supplement: S2 Appendix. — (DOCX) [file pone.0117283.s002.docx]

**Supplementary Information 2** Bayesian models evaluated in the chronosequence study in 2006 and 2009. Response variables are measured on a per quadrat basis, and all cover values are raw values that may sum to greater than 1. Shoreline change rate refers to the distance per year that a shoreline of a particular transect advances towards the coast. The dominant *Ammophila* species refers to transects dominated by either *A. arenaria* or *A. breviligulata*. Chronosequence age refers to the time since the bare ground under a quadrat was formed. Dune gradient refers to the standardized index along a dune cross-section where quadrat occurs.

| Response | Error Structure | Model |
| --- | --- | --- |
| *Ammophila* cover (logit transformed) | Gaussian | ~ (1\|transect) + shoreline change rate + dominant *Ammophila* species + chronosequence age + chronosequence age^2^ + dune gradient + dune gradient^2^ + dominant *Ammophila* × chronosequence age, + dominant *Ammophila* × chronosequence age^2^ + dominant *Ammophila* × dune gradient + dominant *Ammophila* × dune gradient^2^ + chronosequence age × dune gradient + dominant *Ammophila* × chronosequence age × dune gradient |
| Native cover  (logit transformed) | Gaussian | ~ (1\|transect) + shoreline change rate + *Ammophila* cover + dominant *Ammophila* species + chronosequence age + chronosequence age^2^ + dune gradient + dune gradient^2^ + dominant *Ammophila* × chronosequence age, + dominant *Ammophila* × *Ammophila* cover + dominant *Ammophila* × chronosequence age^2^ + dominant *Ammophila* × dune gradient + dominant *Ammophila* × dune gradient^2^ + chronosequence age × dune gradient + dominant *Ammophila* × chronosequence age × dune gradient |
| Species Richness | Poisson |  |
