# Supplementary material for: Invasive Congeners Differ in Successional Impacts across Space and Time
Source: PLoS One. 2015 Feb 6;10(2):e0117283. doi: 10.1371/journal.pone.0117283 (PMC4319750; doi:10.1371/journal.pone.0117283)

**Supplementary Information 4** Parameter estimates and 95% confidence intervals based on the posterior distribution of a hierarchical Bayesian model of (A) native cover and (B) species richness along chronosequence age and dune gradient in 2006 and 2009. Estimates are standardized for comparisons, and CIs that did not contain zero (shown as dashed vertical line) were interpreted as significant. Model parameters converged (Gelman-Rubin Diagnostic Statistic < 1.01) See Appendix S2 for full model.


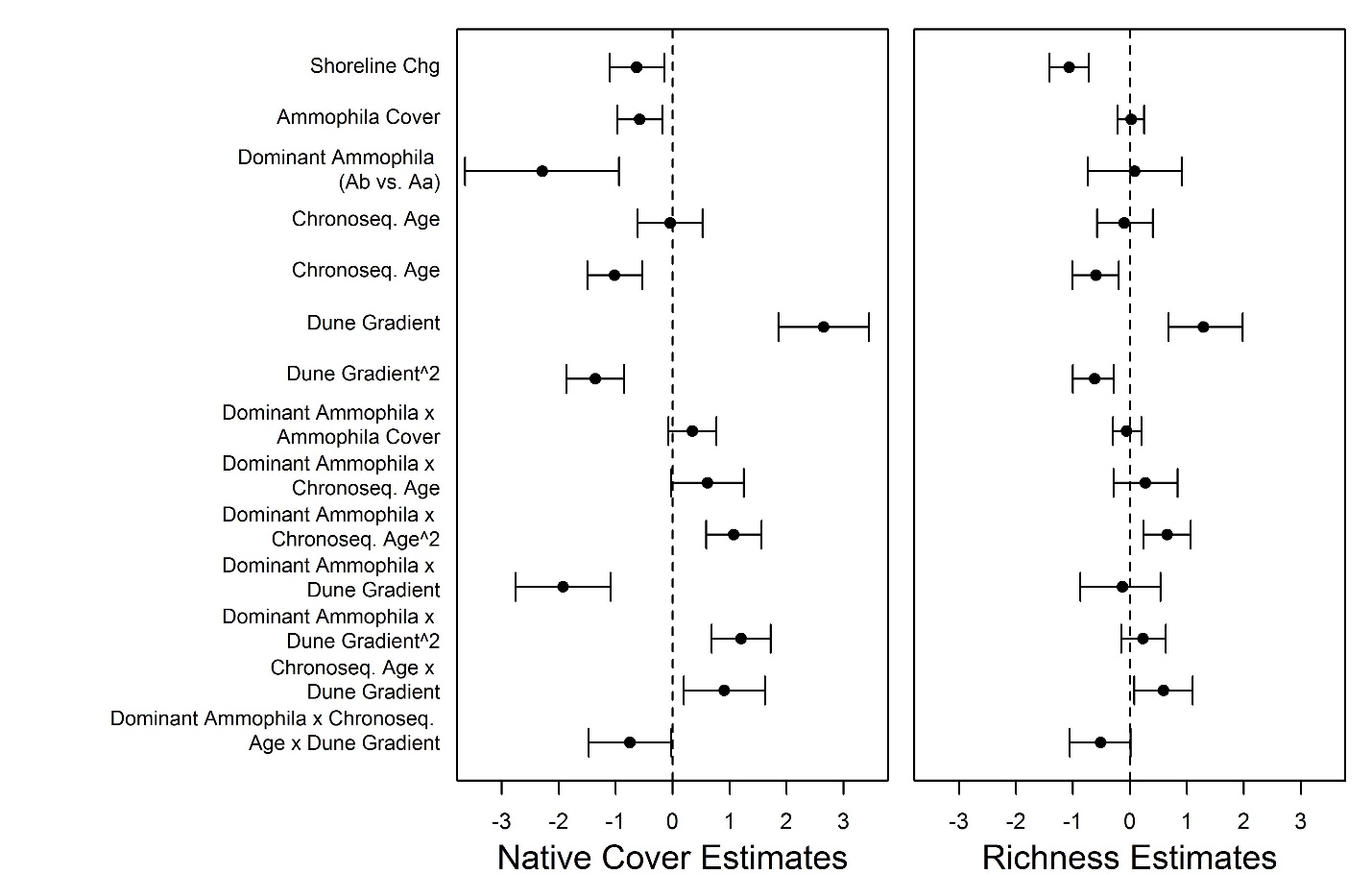

Supplement: S4 Appendix. — (DOCX) [file pone.0117283.s004.docx]
