# Supplementary material for: Invasive Congeners Differ in Successional Impacts across Space and Time
Source: PLoS One. 2015 Feb 6;10(2):e0117283. doi: 10.1371/journal.pone.0117283 (PMC4319750; doi:10.1371/journal.pone.0117283)
Supplement: S5 Appendix. — (DOCX) [file pone.0117283.s005.docx]

**Supplementary Information 5** Effects of *Ammophila* community type and dune gradient on soil properties in 2012 using ANOVA. Dunes were either dominated by *A. arenaria* or *A. breviligulata*. Dune location was categorical and represented the toe, crest, and heel of foredunes.

|  |  | %C | | %N |  | P |  | K |  | Na |  | Organic Matter | | Cation Exchange Capacity | | pH | |
| --- | --- | --- | --- | --- | --- | --- | --- | --- | --- | --- | --- | --- | --- | --- | --- | --- | --- |
| *Source* | *d.f.* | *F* | *p* | *F* | *p* | *F* | *p* | *F* | *p* | *F* | *p* | *F* | *p* | *F* | *p* | *F* | *p* |
| *Ammophila* Community  (Ab vs. Aa) | 1 | 38.174 | 0.000 | 6.312 | 0.015 | 51.514 | 0.000 | 106.653 | 0.000 | 1.009 | 0.319 | 9.910 | 0.003 | 147.120 | 0.000 | 0.760 | 0.387 |
| Location | 2 | 15.248 | 0.000 | 0.371 | 0.691 | 0.393 | 0.677 | 13.158 | 0.000 | 20.893 | 0.000 | 1.943 | 0.153 | 1.415 | 0.251 | 29.999 | 0.000 |
| *Ammophila* Community × Location | 2 | 1.613 | 0.208 | 3.103 | 0.053 | 0.935 | 0.398 | 2.914 | 0.062 | 0.924 | 0.403 | 4.223 | 0.019 | 2.369 | 0.103 | 3.690 | 0.031 |
| Residuals | 57 |  |  |  |  |  |  |  |  |  |  |  |  |  |  |  |  |
