# Supplementary material for: Invasive Congeners Differ in Successional Impacts across Space and Time
Source: PLoS One. 2015 Feb 6;10(2):e0117283. doi: 10.1371/journal.pone.0117283 (PMC4319750; doi:10.1371/journal.pone.0117283)

**Supplementary Information 6** Soil properties in *Ammophila* species dunes along dune gradient. Solid lines show *A. breviligulata* dune soils, dashed lines show *A. arenaria* dune soils. Error bars showed mean ± 1 S.E. Plots show (a) percent C, (b) percent N, (c) concentration of P, (d) concentration of K, (e) concentration of Na, (f) organic matter, (g) cation exchange capacity, and (h) pH. See Table 1 for significance.


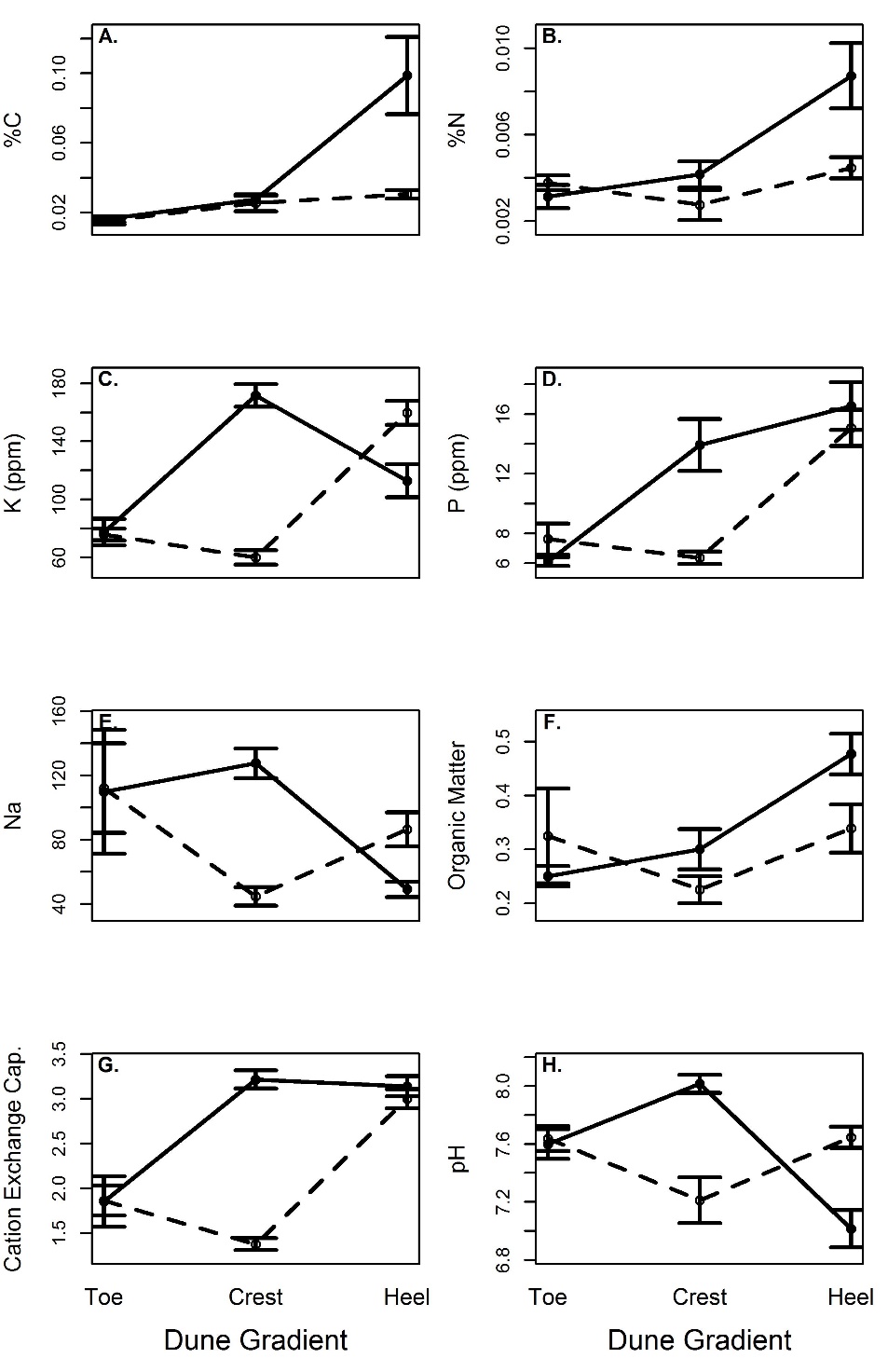

Supplement: S6 Appendix. — (DOCX) [file pone.0117283.s006.docx]
